# Supplementary material for: Immune Checkpoint Inhibitors and Pregnancy: Analysis of the VigiBase® Spontaneous Reporting System
Source: Cancers (Basel). 2022 Dec 28;15(1):173. doi: 10.3390/cancers15010173 (PMC9818632; doi:10.3390/cancers15010173)
Supplement: Supplementary file 1 [file cancers-15-00173-s001.zip › cancers-2042593-supplementary.pdf]

cancers

MDPI

Supplementary Materials

Immune Checkpoint Inhibitors and Pregnancy: Analysis of the VigiBase® Spontaneous Reporting System

Roberta Noseda, Laura Müller, Francesca Bedussi, Michele Fusaroli, Emanuel Raschi and Alessandro Ceschi

Table S1. Summary of the safety reports included in the study.

Time of exposure to ICIs	Number of safety reports	Number of safety reports with fetal events	Suspected ICI(s)	Indication	Reported pregnancy-related adverse events
Before pregnancy	3	3	2 pembrolizumab<br>1 nivolumab	2 melanoma<br>1 lymphoma	Fetal<br>3 abortion (spontaneous or induced)<br>Maternal<br>1 antiphospholipid syndrome/autoimmune disorder<br>1 abdominal distension<br>1 chest pain<br>1 fatigue<br>1 anxiety<br>1 triiodothyronine increased<br>1 lung disorder/dyspnoea<br>1 insomnia<br>1 pruritus
During pregnancy	77	26	31 nivolumab<br>21 pembrolizumab<br>13 ipilimumab and nivolumab<br>11 ipilimumab<br>1 atezolizumab	23 melanoma<br>10 lymphoma<br>4 renal cell carcinoma<br>3 colon cancer<br>3 lung cancer<br>1 breast cancer<br>1 gestational trophoblastic tumor<br>1 glioma<br>31 not reported	Fetal<br>17 premature baby/delivery<br>5 abortion (spontaneous or induced)<br>5 fetal growth restriction<br>2 stillbirth/fetal death<br>1 motor development delay<br>1 fetal distress syndrome<br>1 neonatal respiratory distress syndrome<br>1 umbilical cord compression/hypoxia<br>1 congenital hand malformation<br>1 hypospadias<br>1 neonatal type 1 diabetes mellitus<br>1 retinopathy of prematurity<br>1 intraventricular haemorrhage neonatal<br>1 congenital hypothyroidism<br>1 placental disorder<br>Maternal<br>3 diarrhoea<br>2 white blood cell count increased/neutropenia<br>1 arhtralgia<br>1 lung disorder<br>1 hypophagia/starvation<br>1 urinary tract infection<br>1 iron deficiency anaemia<br>1 nausea<br>1 C-reactive protein increased<br>1 ketoacidosis

					1 abdominal pain<br>1 HELLP syndrome
Exposure via father	12	9	7 nivolumab<br>2 ipilimumab and nivolumab<br>2 pembrolizumab<br>1 atezolizumab	2 renal cell carcinoma<br>1 melanoma<br>9 not reported	Fetal<br>5 abortion (spontaneous or induced)<br>2 foetal growth restriction/small for gestational age<br>1 congenital pulmonary valve disorder<br>1 placental infarction
Unspecified time of exposure	11	8	4 pembrolizumab<br>3 atezolizumab<br>3 nivolumab<br>1 ipilimumab	3 lymphoma<br>2 melanoma<br>1 pericardial mesothelioma<br>1 renal cell carcinoma<br>1 alveolar soft part sarcoma<br>3 not reported	Fetal<br>6 abortion (spontaneous or induced)<br>1 premature delivery<br>1 neonatal respiratory distress syndrome<br>Maternal<br>1 pre-eclampsia<br>1 diabetes mellitus<br>1 hypophisitis<br>1 nausea<br>1 chest pain<br>1 fatigue<br>1 abdominal pain<br>1 pruritus

Abbreviations: ICI immune checkpoint inhibitor.

Table S2. Safety reports associated with immune checkpoint inhibitors reporting pregnancy-related outcomes and having concomitant drugs as potential confounding factors.

**Safety report progressive number**	**Suspected ICI(s)**	**Indication**	**Reported pregnancy-related event(s)**	**Concomitant drugs**	**Reproductive risk according to Reprotox® database (accessed 25 October 2022)**	**References**
Safety report #1	nivolumab	Not reported	Foetal exposure during pregnancy<br>Foetal distress syndrome<br>Foetal growth restriction<br>Premature baby<br>Congenital hand malformation	Azathioprine<br>Sertraline<br>Levothyroxine<br>Sumatriptan	Azathioprine: "Environmental hazards to human reproduction and development".<br>"Quick take: There are case reports of normal human pregnancy outcome after exposure to azathioprine. Most of the small controlled studies on the subject are consistent with a lack of azathioprine-associated risk of congenital anomalies after pregnancy exposure; however, one study found an increase in atrial and ventricular septal defects and in preterm delivery. Neonatal hematologic and immune impairment were reported in some exposed infants. It is not clear if growth restriction and shortened gestation are due to azathioprine, concomitant medications, or the underlying maternal illness."<br>Sertraline: "Environmental hazards to human reproduction and development".<br>"Quick take: Based on experimental animal studies and human experience, sertraline is not expected to increase the risk of congenital anomalies. Human studies have inconsistently reported associations of sertraline use during pregnancy and various defects in the offspring. Use of serotonin re-uptake inhibitors late in pregnancy has been associated with a mild transient neonatal syndrome of central nervous system, motor, respiratory, and gastrointestinal signs. Use of fluoxetine, sertraline, or paroxetine was associated with an increased risk of neonatal pulmonary hypertension in some but not all studies. Long-term	-

					neurodevelopmental studies suggest that antenatal exposure to fluoxetine, sertraline, or paroxetine does not adversely affect outcome."<br>Sumatriptan: "Environmental hazards to human reproduction and development".<br>"Quick take: Experimental animal studies have not shown abnormal embryo development with sumatriptan exposures close to those encountered in therapy. Human experience with sumatriptan exposure during pregnancy has been reassuring."	
Safety report #2	Combination of nivolumab and ipilimumab	Not reported	Exposure via body fluid<br>Placental infarction<br>Normal newborn	Colecalciferol<br>Denosumab	Colecalciferol: "Environmental hazards to human reproduction and development".<br>"Quick take: Vitamin D is an essential nutrient. The Dietary Reference Intake during pregnancy and lactation is 15 mcg (600 IU); however, some investigators have recommended much higher intakes. Excess vitamin D has not been associated with a syndrome of congenital anomalies. Transient neonatal hypercalcemia has been identified in at least one case report."<br>Denosumab: "Environmental hazards to human reproduction and development".<br>"Quick take: Denosumab is avoided during pregnancy due to impaired fetal viability and interference in monkeys with bone and lymph node development. We did not locate human data."	-
Safety report #3	pembrolizumab	Not reported	Foetal exposure during pregnancy<br>Premature baby	Dexamethasone<br>Morphine<br>Oxycodone<br>Fluconazole	NA$	[36–39]
Safety report #4	nivolumab and ipilimumab in regimen not definable	Not reported	Foetal exposure during pregnancy<br>Premature baby<br>Lung disorder<br>C-reactive protein increased<br>White blood cell count increased	Betamethasone	NA$	[36]
Safety report #5	nivolumab and ipilimumab in regimen not definable	Not reported	Foetal exposure during pregnancy<br>Foetal growth restriction<br>Premature baby	Betamethasone	NA$	[36]
Safety report #6	nivolumab and ipilimumab in regimen not definable	Not reported	Foetal exposure during pregnancy<br>Foetal growth restriction<br>Premature baby	Betamethasone	NA$	[36]
Safety report #7	nivolumab and ipilimumab in	Malignant melanoma	Maternal exposure during pregnancy	Betamethasone	NA$	[36]

	regimen not definable		Premature delivery<br>Iron deficiency anaemia			
Safety report #8	nivolumab and ipilimumab in regimen not definable	Malignant melanoma	Maternal exposure during pregnancy<br>Premature delivery	Betamethasone	NA$	[36]
Safety report #9	nivolumab	Not reported	Foetal exposure during pregnancy<br>Umbilical cord compression<br>Hypoxia	Doxorubicin<br>Vinblastine<br>Dacarbazine<br>Bleomycin	NA*	-
Safety report #10	nivolumab	Malignant melanoma	Maternal exposure during pregnancy<br>Normal newborn	Insulin<br>Ramipril	NA*	-

$ Concomitant drug(s) were not confounding factors rather were more likely used to optimize the management of premature neonates. * The reported pregnancy-related outcomes did not have any relation with the concomitant drugs. *Abbreviations:* NA not applicable.
